# Functional and Structural Characteristics of Tumor Angiogenesis in Lung Cancers Overexpressing Different VEGF Isoforms Assessed by DCE- and SSCE-MRI

**DOI:** 10.1371/journal.pone.0016062

**Published:** 2011-01-20

**Authors:** Ang Yuan, Chien-Yuan Lin, Cheng-Hung Chou, Chia-Ming Shih, Chih-Yuan Chen, Hao-Wei Cheng, Yi-Fang Chen, Jeremy J. W. Chen, Jyh-Horng Chen, Pan-Chyr Yang, Chen Chang

**Affiliations:** 1 Department of Internal Medicine, National Taiwan University Hospital, Taipei, Taiwan, Republic of China; 2 Institute of Biomedical Sciences, Academia Sinica, Taipei, Taiwan, Republic of China; 3 Institutes of Biomedical Sciences and Molecular Biology, National Chung Hsing University, Taichung, Taiwan, Republic of China; 4 Interdisciplinary MRI/MRS Lab, Institute of Biomedical Electronics and Bioinformatics, National Taiwan University, Taipei, Taiwan, Republic of China; National Cancer Institute, United States of America

## Abstract

The expressions of different vascular endothelial growth factor (VEGF) isoforms are associated with the degree of tumor invasiveness and the patient's prognosis in human cancers. We hypothesized that different VEGF isoforms can exert different effects on the functional and structural characteristics of tumor angiogenesis. We used dynamic contrast-enhanced MRI (DCE-MRI) and steady-state contrast-enhanced MRI (SSCE-MRI) to evaluate *in vivo* vascular functions (e.g., perfusion and permeability) and structural characteristics (e.g., vascular size and vessel density) of the tumor angiogenesis induced by different VEGF isoforms (VEGF121, VEGF165, and VEGF189) in a murine xenograft model of human lung cancer. Tumors overexpressing VEGF189 were larger than those overexpressing the other two VEGF isoforms. The *K* ^trans^ map obtained from DCE-MRI revealed that the perfusion and permeability functions of tumor microvessels was highest in both the rim and core regions of VEGF189-overexpressing tumors (*p*<0.001 for both tumor rim and core). The relative vessel density and relative vessel size indexes derived from SSCE-MRI revealed that VEGF189-overexpressing tumors had the smallest (*p*<0.05) and the most-dense (*p*<0.01) microvessels, which penetrated deeply from the tumor rim into the core, followed by the VEGF165-overepxressing tumor, whose microvessels were located mainly in the tumor rim. The lowest-density microvessels were found in the VEGF121-overexpressing tumor; these microvessels had a relatively large lumen and were found mainly in the tumor rim. We conclude that among the three VEGF isoforms evaluated, VEGF189 induces the most densely sprouting and smallest tumor microvessels with the highest *in vivo* perfusion and permeability functions. These characteristics of tumor microvessels may contribute to the reported adverse effects of VEGF189 overexpression on tumor progression, metastasis, and patient survival in several human cancers, including non-small cell lung cancer, and suggest that applying aggressive therapy may be necessary in human cancers in which VEGF189 is overexpressed.

## Introduction

Angiogenesis is required for tumor growth and metastasis [1], [2], and it has been shown that high tumor angiogenesis activity is associated with advanced tumor growth, distant metastases, and an adverse prognosis in human cancers [3], [4]. Vascular endothelial growth factor (VEGF) is a potent angiogenesis factor under both physiological and pathological conditions, and can induce tumor angiogenesis [5], [6]. The human VEGF gene (*VEGF-A*), which is located on chromosome 6p, contains eight exons [7]. Alternative splicing of the VEGF gene gives rise to multiple isoforms. Three major VEGF-A isoforms (VEGF121, VEGF165, and VEGF189) are expressed in a variety of human tissues and tumor specimens [5], [8], and have differential biochemical properties and biological functions in physiological angiogenesis [9]–[13].

The biological functions of different VEGF-A isoforms in pathological angiogenesis, such as tumor angiogenesis, are still unclear. Several studies, including our previous study, have shown that the expression of VEGF189 in a tumor is strongly associated with a high tumor microvessel count, cancer metastasis, and short patient survival in several human cancers [14]–[16], and with the high xenotransplantability of several human cancer cells in mice [17]. However, the mechanisms underlying these activities remain unknown. Few studies have evaluated the roles of the different major VEGF isoforms in determining the functions of the tumor microvasculature and in facilitating tumorigenesis and tumor metastasis via tumor angiogenesis.

Dynamic contrast-enhanced MRI (DCE-MRI) with the utilization of T1 contrast medium Magnevist (Gd-DTPA, MW: 938 Dalton), is able to provide a vascular functional parameter, the vascular transfer constant (*K* ^trans^), which gives a measure of the perfusion and permeability of vessels [18]–[21]. In addition, steady-state contrast-enhanced MRI (SSCE-MRI) in combination with T2 agent, Resovist (super paramagnetic iron oxide (SPIO) particles, size: 45–60 nm), can give structural information on microvasculature, namely the relative vessel density index (rVDI) and the relative vessel size index (rVSI) [22]–[26]. MRI is therefore rapidly emerging as a promising method for assessing vascular perfusion and permeability, and microvessel density (MVD) and size *in vivo* in several human cancers [27]–[30].

We hypothesized that different VEGF isoforms can induce tumor angiogenesis with different biological functions. Therefore, we used DCE- and SSCE-MRI to evaluate the functional and structural characteristics of tumor angiogenesis in lung cancers overexpressing one of three different single VEGF isoforms (VEGF121, VEGF165, or VEGF189) in a murine xenograft model. The aim of this study was to test whether *K* ^trans^, rVDI, and rVSI of tumor angiogenesis differed among lung tumors overexpressing one of three different VEGF isoforms. The results will help to elucidate the role of different VEGF isoforms in inducing tumor angiogenesis, the interaction between the structure and function of tumor angiogenesis, and the mechanisms underlying the association between the expression of a specific VEGF isoform in a tumor and the patient's clinical outcome in human cancers.

## Results

### Generation of stable CL1-0 cell lines expressing different VEGF isoforms

**Figure 1:**
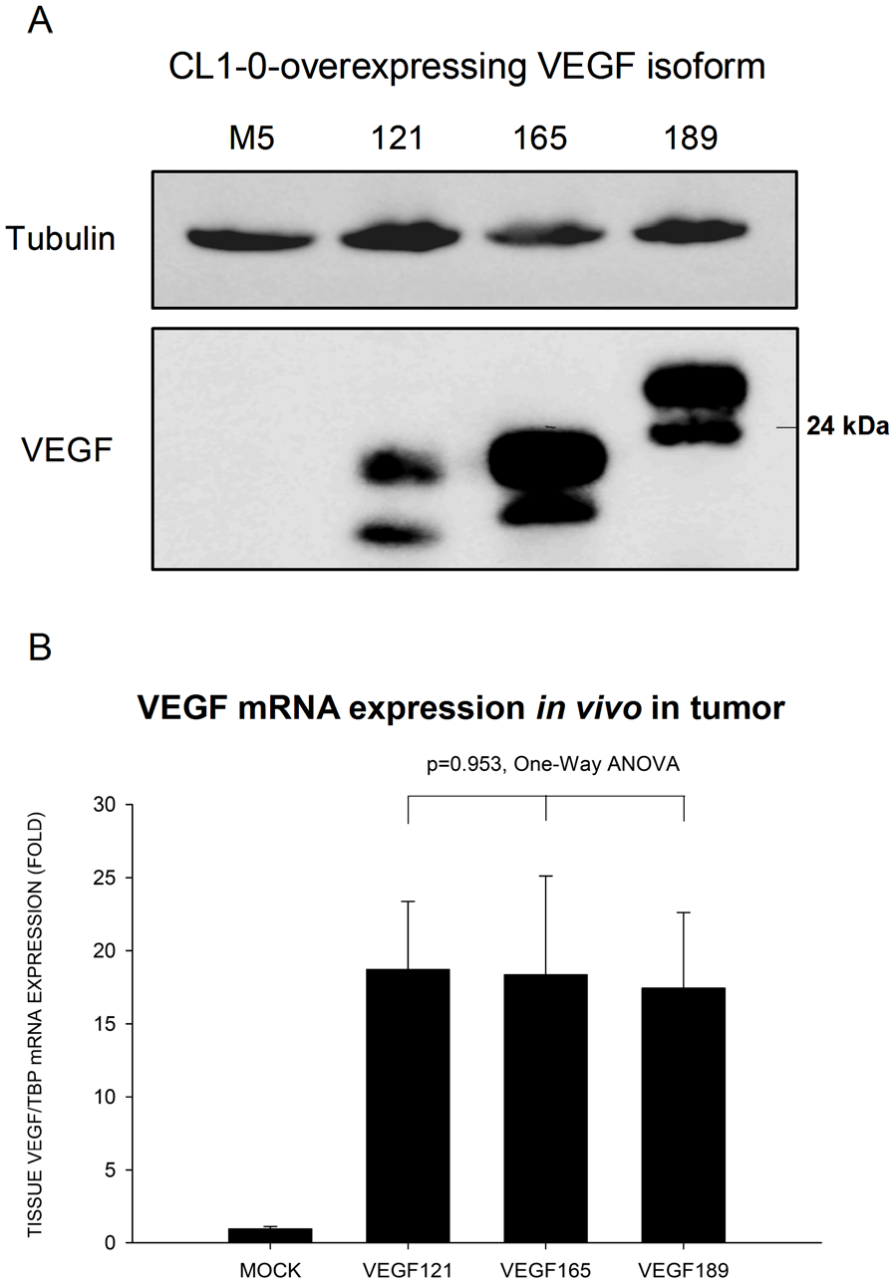
Western blots of VEGF isoforms proteins expression in lung cancer cells and real-time quantitative RT-PCR of VEGF isoform mRNA expression in tumor implants. A. Western blots of VEGF isoform protein expression in cell lysate of human CL1-0 lung cancer cells transfected with single different VEGF isoform constructs. Each VEGF isoform protein comprised one glycosylated (upper) and one unglycosylated (lower) protein. Tubulin was used as an internal control. B. Quantification of VEGF mRNA expression *in vivo* in the tumor implants by real-time quantitative reverse transcription-PCR. The expression level of VEGF isoform was similar among CL1-0 lung cancer cell lines overexpressing one of three VEGF isoforms (p = 0.953, one-way ANOVA).

The parent lung cancer cell line CL1-0 was stably transfected with VEGF121, VEGF165, or VEGF189 isoform cDNA or the empty vector (mock transfected). About 20 clones of each VEGF-isoform-expressing cell were analyzed, and one panel (i.e., VEGF121-3, VEGF165-C12, and VEGF189-A3) was ultimately selected for experiments, as they showed similar levels of VEGF isoform expression. The recombinant VEGF isoform mRNA expressed by each clone was confirmed by real-time quantitative RT-PCR. The recombinant protein expressed was also confirmed by Western blotting (Fig. 1A), with the expected molecular weights being 18 kDa (VEGF121), 23 kDa (VEGF165), and 26 kDa (VEGF189). The amount of each VEGF isoform in the culture supernatant, as determined by ELISA, was 1.14×10^2^−2.9×10^2^ ng/cell in a 48-h period. The VEGF isoforms expression level *in vivo* in the implanted tumors determined by real-time quantitative RT-PCR was similar among tumors overexpressing one of three VEGF isoform **(p = 0.953, one-way ANOVA)** (Fig. 1B).

### Tumor volumes and growth curves of the VEGF-isoform-overexpressing tumors, as measured by T2WI

**Figure 2:**
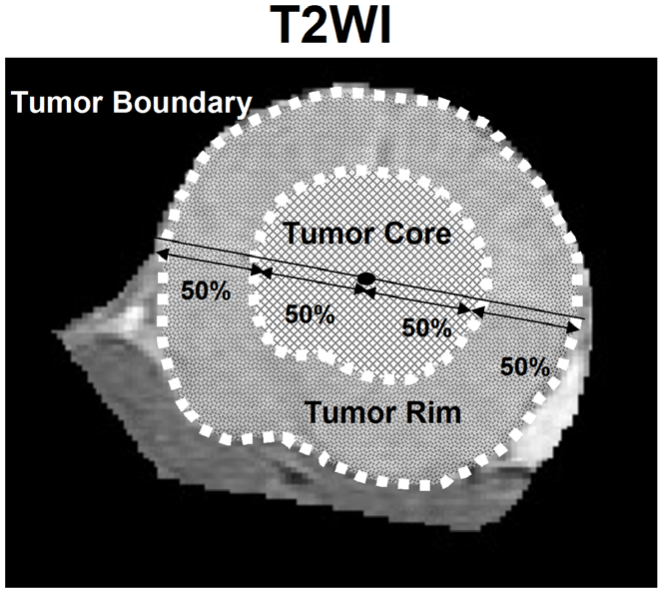
Determining the core and rim regions of tumors on T2WI for analyzing quantitative maps (*K* ^trans^, rVSI, and rVDI).

**Figure 3:**
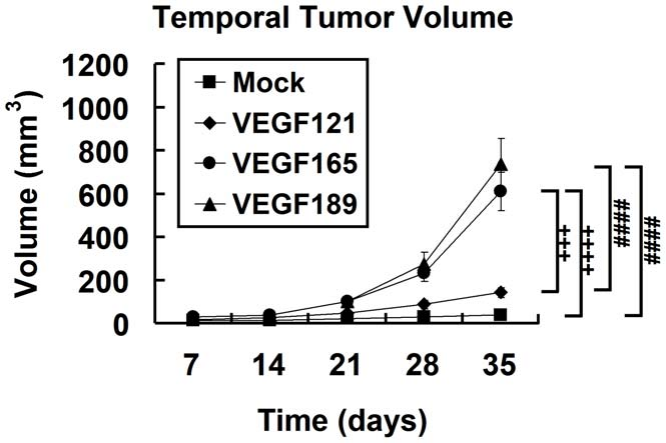
Growth curves of different VEGF isofrom overexpressing tumors and the mock tumors. Tumor growth of CL1-0 cancer cells overexpressing one of three VEGF isoforms at different time points in SCID mice, estimated using T2WI. #, VEGF189 versus VEGF121 and the mock tumors; +, VEGF165 versus VEGF121 and the mock tumors; *, comparison between different isoform-expressing and mock tumors. Differences between VEGF-overexpressing tumors and mock tumors were significant at the *p*<0.05 (one symbol), *p*<0.01 (two symbols), *p*<0.001 (three symbols), and *p*<0.0001 (four symbols) levels.

The volumes of VEGF121-, VEGF165-, and VEGF189- overexpressing tumors, as measured on T2-weighted images (Fig. 2) increased from day 7 to day 35 after inoculation, whereas mock tumors did not show any significant increase in tumor volume (The mock tumor indicates the tumor derived from CL1-0 lung cancer cell line transfected with an empty vector). The tumor growth curve showed that the VEGF189- and VEGF165- overexpressing tumors grew faster than the others (*n* = 6, *p*<0.0001 by two-way ANOVA; all *p* values <0.001 by Fisher's *post hoc* test; Fig. 3). The growth curve also showed that the VEGF189- and VEGF165-overexpressing tumors grew rapidly and exponentially after day 21 (*p*<0.05, by Fisher's *post hoc* test; Fig. 3).

At the end of day 35, the volumes of the tumor xenografts were 141.1±22.4 mm^3^, 610.8±87.8 mm^3^, and 738.0±116.1 mm^3^ for the VEGF121-, VEGF165-, and VEGF189-overexpressing tumors, respectively, while that of the mock tumor xenograft was 37.4±7.9 mm^3^. The volumes of the VEGF165- and VEGF189-overexpressing tumors on day 35 were significantly larger than those of the mock-transfected and VEGF121-overexpressing tumors (Fig. 3; all *p*<0.001 by Fisher's *post hoc* test). The volume of the VEGF189-overexpressing tumor tended to be larger than that of the VEGF165-overexpressing tumor, but the difference did not reach statistical significance.

### Perfusion and permeability of tumor microvessels evaluated by the *K* ^trans^ map on DCE-MRI

**Figure 4:**
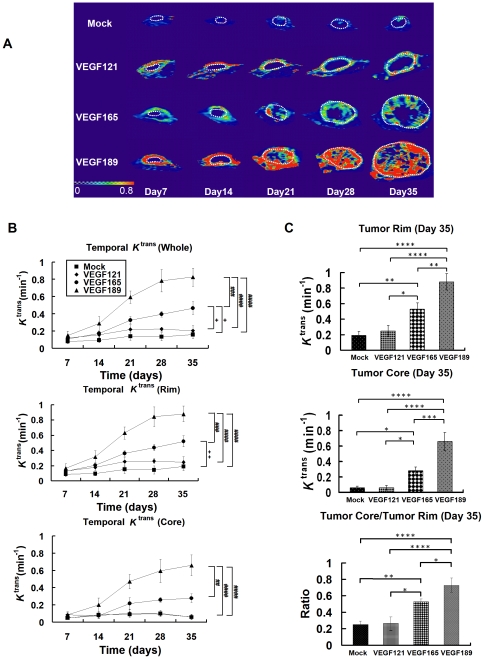
The temporal *K* ^trans^ maps and values of different VEGF isofrom overexpressing tumors and the mock tumors. *In vivo* temporal *K* ^trans^ map and quantitative curve for tumor xenografts of CL1-0 cancer cells overexpressing one of three VEGF isoforms at different time points, evaluated by DCE-MRI. (A) Representative *K* ^trans^ color map in the different VEGF-overexpressing and mock-transfected tumors (within dotted circle) at different times, from day 7 to day 35 postimplantation. The color ranged from blue (0/min, lowest *K* ^trans^) to red (0.8/min, highest *K* ^trans^). B) Quantitative analysis of *K* ^trans^ values over time in the regions of the whole tumor (upper), tumor rim (middle), and tumor core (lower). #, VEGF189 versus other isoforms and the mock tumors; +, VEGF165 versus VEGF121 and mock tumors. Differences between VEGF-overexpressing tumors and mock tumors were significant at the *p*<0.05 (one symbol), *p*<0.01 (two symbols), *p*<0.001 (three symbols), and *p*<0.0001 (four symbols) levels. (C) *K* ^trans^ values on day 35 in the tumor rim (upper) and tumor core (middle), and the ratio of *K* ^trans^ values between the tumor core and tumor rim (lower). Differences between VEGF-overexpressing tumors and mock tumors were significant at the *p<0.05, **p<0.01, ***p<0.001, and ****p<0.0001 levels.

Figure 4A shows the temporal *K* ^trans^ map for the three VEGF-isoform-overexpressing tumors from day 7 to day 35. Various angiogenic patterns of *K* ^trans^ were observed in the different VEGF-overexpressing and mock tumors. On day 35, the *K* ^trans^ signal was observed mainly in the tumor rim in the mock-transfected and VEGF121-overexpressing tumors, while in the VEGF165-overexpressing tumor, the *K* ^trans^ signal was located mainly in the rim, with some in the core. A substantial amount of signal extending from the rim to the core was noted in the VEGF189-overexpressing tumor.

The temporal change in *K* ^trans^ values in the whole, rim, and core of tumor increased significantly after day 14 in the VEGF189- and VEGF165-overexpressing tumors, while there was no significant increase in *K* ^trans^ values for the mock-transfected and VEGF121-overexpressing tumors (Fig. 4B). The temporal *K* ^trans^ curves were significantly higher in the VEGF189-overexpressing tumors in the whole tumor (Fig. 4B, upper), tumor rim (Fig. 4B, middle), and tumor core (Fig. 4B, lower) than in the others (all *p*<0.001 by Fisher's *post hoc* test).

On day 35, the *K* ^trans^ values at the rim and core of the tumors were 0.25±0.07/min and 0.06±0.03/min, respectively, for VEGF121-overexpressing tumors, 0.53±0.08/min and 0.28±0.05/min for VEGF165-overexpressing tumors, 0.88±0.1/min and 0.66±0.12/min for VEGF189-overexpressing tumors, and 0.19±0.05/min and 0.06±0.02/min for mock tumor xenografts (Fig. 4C). *K* ^trans^ on day 35 was significantly higher in the VEGF189-overexpressing tumors than in the other three types (all *p*<0.01 for the rim and *p*<0.001 for the core by Fisher's *post hoc* test; Fig. 4C, upper, middle). The core/rim ratio of *K* ^trans^ in VEGF189-overexpressing tumors on day 35 was 0.73±0.09, which was significantly higher than those in the VEGF165- (0.53±0.03) and VEGF121- (0.26±0.08) overexpressing tumors and in the mock tumor xenografts (0.25±0.05; *p*<0.05, *p*<0.0001, and *p*<0.0001, respectively, by Fisher's *post hoc* test). This indicates that the signal of the *K* ^trans^ map was distributed most deeply from the rim to the core regions of the VEGF189-overexpressing tumors (Fig. 4C, lower).

### Vascular structural characteristics of tumor angiogenesis assessed by the rVDI and rVSI on SSCE-MRI

**Figure 5:**
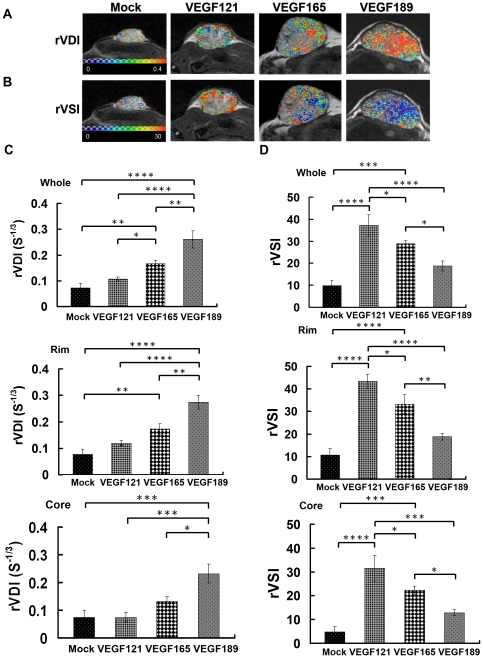
The rVDI and rVSI maps and values of different VEGF isofrom overexpressing tumors and the mock tumors. *In vivo* rVDI and rVSI maps and quantitative curves for tumor xenografts of CL1-0 cancer cells overexpressing one of three VEGF isoforms, evaluated by SSCE-MRI. Representative high-resolution maps of the (A) rVDI and (B) rVSI in the different VEGF-overexpressing and mock tumors on day 36 after tumor implantation. In rVDI map, the color ranged from blue (0 S^-1/3^, lowest rVDI) to red (0.4 S^-1/3^, highest rVDI). In rVSI map, the color ranged from blue (0, lowest rVSI) to red (30, highest rVSI).Quantitative analysis of (C) rVDI and (D) rVSI in the whole tumor (upper), tumor rim (middle), or tumor core (lower). Differences between VEGF-overexpressing tumors and mock tumors were significant at the *p<0.05, **p<0.01, ***p<0.001, and ****p<0.0001 levels.

Figure 5A and B show representative maps of rVDI and rVSI, respectively, in the mock and different VEGF-overexpressing tumors on day 36. The rVDI map shows only a few scattered vessel density signals in the tumor rim in a mock-transfected tumor. The VEGF189-overexpressing tumor shows a high density of microvessels distributed from the rim to the core of the tumor. In contrast, the VEGF121-overexpressing tumor exhibits a low density of microvessels that is limited to the rim, and the VEGF165-overexpressing tumor has a mixed low and high density of microvessels that are distributed mainly in the rim, with some in the core (Fig. 5A). As shown in the upper panel of Fig. 5C, the rVDI of the whole tumor was highest in VEGF189-overexpressing tumors (0.26±0.03/s^1/3^), intermediate in VEGF165-overexpressing tumors (0.17±0.01/s^1/3^), and lowest in VEGF121-overexpressing (0.11±0.01/s^1/3^) and mock-transfected (0.07±0.02/s^1/3^) tumors (all *p*<0.01 by Fisher's *post hoc* test). In both the rim and core, VEGF189-overexpressing tumors exhibited the highest rVDI values among all VEGF-isoform-overexpressing and mock-infected tumors (Fig. 5C, middle and lower panels).

The rVSI map shows a few signals in the tumor rim in the mock tumor. The rVSI map also indicates that the tumor microvessels were almost all large vessels in the VEGF121-overexpressing tumors (in the rim), mixed large and small in the VEGF165-overexpressing tumors (mainly in the rim with some in the core), and almost all small in the VEGF189-overexpressing tumors (from the rim to the core; Fig. 5B).

As shown in the upper panel of Fig. 5D, the rVSI of the whole tumor was highest in the VEGF121-overexpressing tumors (37.24±4.88), intermediate in the VEGF165-overexpressing tumors (28.84±1.46), and lowest in the VEGF189-overexpressing (18.82±2.27) and mock (9.84±2.25) tumors (all *p*<0.05 by Fisher's *post hoc* test). In both the rim and core, the VEGF189-overexpressing tumor had the lowest rVSI among the VEGF-isoform-overexpressing tumors (Fig. 5D, middle and lower panels).

### Angiogenesis phenotype of tumors overexpressing different VEGF isoforms by immunohistochemical staining

**Figure 6:**
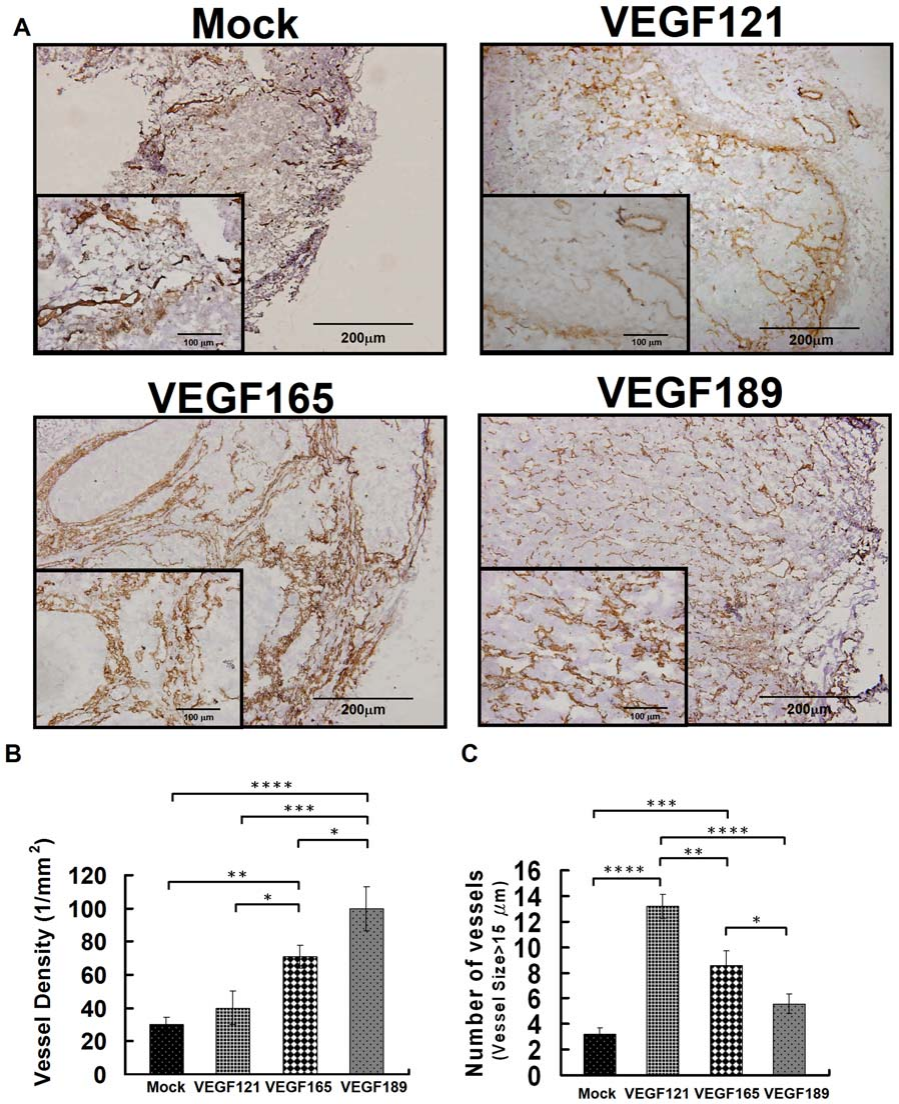
The immunohistochemical staining of microvessels of different VEGF isofrom overexpressing tumors and the mock tumors. Angiogenesis phenotypes of microvessels evaluated by immunohistochemical staining of tumor xenografts overexpressing different VEGF isoforms. (A) Immunohistochemical staining of tumor microvessels (brown color, ×100 in main panel, ×400 in the inset) in tumor xenografts. (B) The microvessel density was highest in the VEGF189-overexpressing tumors, intermediate in the VEGF165-overexpressing tumors, and lowest in the VEGF121-overexpressing tumors. (C) The number of vessels with a diameter larger than 15 µm per tumor section in the tumor xenograft was highest in the VEGF121-overexpressing tumors, intermediate in the VEGF165-overexpressing tumors, and lowest in the VEGF189-overexpressing tumors. Differences between VEGF-overexpressing and mock tumors were significant at the *p<0.05, **p<0.01, ***p<0.001, and ****p<0.0001 levels.

At low power (Fig. 6A, ×100), the immunohistochemical staining revealed a small number of microvessels distributed in the periphery of the tumor nests of the mock tumors, and denser tumor microvessels distributed mainly at the periphery of the tumor or tumor nests in the VEGF121- and VEGF165-overexpressing tumors. In the VEGF189-overexpressing tumors, very dense microvessels were seen at both the rim and in the core.

At high power (Fig. 6A, ×400, insert), VEGF121-overexpressing tumors had more large microvessels with dilated lumens in addition to the small-lumen microvessels, and VEGF165-overexpressing tumors had intermediate-sized microvessels. In contrast, in the VEGF189-overexpressing and the mock-transfected tumors, almost all of the microvessels had a small lumen.

### MVD and number of large microvessels in tumor xenografts on immunohistochemical staining

As shown in Fig. 6B **,** the MVD measured from immunohistochemical staining of CD31 was highest in VEGF189-overexpressing tumors (100.00±13.30/mm^2^), intermediate in VEGF165-overexpressing tumors (70.66±6.84/mm^2^), and lowest in VEGF121-overexpressing (40.07±10.15/mm^2^) and mock-transfected (29.98±4.24/mm^2^) tumors (all *p*<0.05 by Fisher's *post hoc* test).

As shown in Fig. 6C, the number of vessels with a diameter larger than 15 µm per tumor section in the tumor xenograft was highest in VEGF121-overexpressing tumors (13.21±0.95/mm^2^), intermediate in VEGF165-overexpressing tumors (8.58±1.11/mm^2^), and lowest in VEGF189-overexpressing (5.58±0.76/mm^2^) and mock-transfected (3.21±0.47/mm^2^) tumors (all *p*<0.05 by Fisher's *post hoc* test).

## Discussion

Much remains to be elucidated about tumor-associated vascular abnormalities; therefore, collecting more complete data regarding changes in angiogenic microvascular structure and function can facilitate the understanding of the mechanisms underlying tumor angiogenesis and thus provide therapeutic implications. The *K* ^trans^, rVDI, and rVSI parameters obtained by DCE- and SSCE-MRI are capable of providing data on vascular perfusion and permeability, vessel density, and vessel size, respectively. To the best of our knowledge, this is the first study to have used contrast-enhanced MRI to evaluate the functional and structural characteristics of tumor microvessels induced by different VEGF isoforms *in vivo*, and to have correlated the functional and structural characteristics of angiogenesis in VEGF-isoform-overexpressing tumors.

Different VEGF isoforms reportedly exhibit different biochemical properties. VEGF121 is a shorter, non-heparin-binding acidic protein and is freely diffusible, whereas VEGF165 and VEGF189 can bind heparin and heparan sulfate proteoglycans (HSPGs) [13]. VEGF189 is frequently associated with the cell surface and extracellular matrix, whereas VEGF165 is both a secreted protein and is HSPG-associated [13]. VEGF165 and VEGF189 each have different affinities for different coreceptors, such as neuropilin-1 and neuropilin-2, and for HSPGs, such as syndecan [14]. Recent studies have shown that different VEGF isoforms can induce tumor angiogenesis exhibiting different morphological characteristics [31]–[34]. VEGF121- and VEGF165-induced tumor microvessels were associated with vasodilation, microvessel eruption, and hemorrhage [32]. In contrast, VEGF189 can induce intense tumor angiogenesis consisting of small, newly formed microvessels [31], [33]. In this study, our data from SSCE-MRI were consistent with these morphological findings. We further demonstrated that different VEGF isoforms can induce microvessels with different *in vivo* vascular functions, and that VEGF189-induced angiogenesis in lung cancers is characterized by the highest perfusion and permeability characteristics. These results suggest that VEGF189 can induce the most-dense and smallest sprouting tumor microvessels that have the highest perfusion and permeability characteristics among the three VEGF-isoform-overexpressing tumors examined.

The precise molecular and biological mechanisms underlying the different morphological and functional characteristics of tumor angiogenesis induced by different VEGF isoforms remain unclear; further investigation is required to clarify this. Some recent investigations have provided possible explanations for these differences. Ruhrberg *et al.* showed that in the *vegf^188/188^* mouse brain, there was an increase in endothelial filopodia extensions, which may lead to increased vascular branching and sprouting angiogenesis [35]. In contrast, VEGF121 was shown to lack the ability to induce sprouting angiogenesis [35], but only coopts preexisting peritumor vessels and induces vasodilation. Our MRI data provide solid evidence of the biological behavioral differences of endothelial cells stimulated by different VEGF isoforms *in vivo* in tumor angiogenesis. There is also evidence that the molecular signaling differs among these isoforms. VEGF165 and VEGF189 can bind to VEGF coreceptors, such as neuropilin-1 and HSPGs, and these bindings can modify VEGF signaling in endothelial cells, controlling the bioavailability of VEGF to VEGF receptors [36]–[38]. The activities of VEGF189 in nuclear translocation [34] and crosstalk of VEGF-signaling pathways with integrins [39] may also affect the biological functions of different VEGF isoforms in tumor angiogenesis. The differential cell biological functions and molecular signal transductions also translate to the different *in vivo* morphological and functional characteristics of tumor angiogenesis induced by different VEGF isoforms in human cancers.

*K* ^trans^ mapping revealed that the *K* ^trans^ of the microvessels were highest in VEGF189-overexpressing tumors. The high *K* ^trans^ value of VEGF189-overexpressing tumors may be attributable to the highest density of microvessels that penetrate deeply from the rim to the core of the tumor, which can provide a high vascular perfusion function. Previous studies showed that VEGF189 can enhance the vascular permeability of skin vessels and proliferating intratumoral vessels, as assessed by Mile's vascular permeability assay and Ig G staining [13], [31]. Our data are consistent with these findings, and suggest that the increasing permeability may contribute to the highest *K* ^trans^ value observed in VEGF189-overexpressing tumors. In addition, our previous report has shown that the blood flow (Fρ), microvessel permeability (PSρ) and the blood volume (Vb) determined by DCE-MRI were all significantly increased in tumor overexpressing VEGF 189 isoform [40]. Tozer *et al.* recently showed that mouse VEGF188 can induce narrow microvessels with the greatest microvessel length, resistance to hemorrhage and resistance to antiangiogenesis agent therapy, but is associated with tumor vascular maturity [34]. This may arise from different cell lines to be used to overexpress VEGF isoform (fibrosarcoma cell line vs lung adenocarcinoma cell line), different method of experiment and different model used. However, both studies showed the different VEGF isoform has different function and this may determine the different characteristics of microvessels in tumors in different organs. Although VEGF121-overexpressing tumors have larger microvessel diameters, as shown by the high rVSI, the corresponding *K* ^trans^ value is low. This may indicate that *K* ^trans^ is dependent mainly on the rVDI rather than the rVSI. The precise relationship between *K* ^trans^ and rVSI or rVDI may be difficult to determine due to the complicated tumor microenvironmental physiology with overexpression of different VEGF isoforms. Nevertheless, *K* ^trans^ has been shown previously to be associated with vessel density. Previous data had also shown that stasis of blood will occur in the dilated and tortuous vascular spaces.

Because VEGF121 is a shortest, non-heparin-binding acidic protein which is freely diffusible, they will be secreted from cell to the outside environment once they are synthesized. The amount of VEGF121 protein remained in cell is supposed to be lower as compared to other VEGF isoforms which have higher proportion of VEGF proteins remaining in the cells. Therefore, the density of VEGF121 protein band in Western blot obtained from cell lysate of CL1-0 cancer cells can not stand for the total amount of VEGF121 proteins synthesized or the amount that are secreted to the environment. We have quantitated the amount of VEGF isoforms that are secreted out of cell into the supernatant by ELISA, and the results showed that the amount of VEGF121 protein in the supernatant was similar to the amount of the other two VEGF isoforms. In addition, we further quantitated the VEGF expression level *in vivo* in the tumor implants resected from SCID mice using real-time quantitative RT-PCR, and the result showed the expression level of VEGF isoform was similar among tumors from CL1-0 lung cancer cell lines overexpressing one of three VEGF isoforms (p = 0.953, one-way ANOVA)(Fig. 1B). Therefore, the differences in tumor growth and angiogenic properties between VEGF isoforms in this study are because of different function of VEGF isoforms, in stead of resulting from the different expression level of VEGF between different VEGF isoforms in tumors.

In our study, the VEGF189-induced angiogenesis had the highest MVD, penetrated deeply from the tumor rim to the tumor core, and had the highest perfusion and permeability functions among three different VEGF-isoform-overexpressing tumor types. However, the tumor size did not differ significantly between VEGF165- and VEGF189-overepxressing tumors. We observed the viability of the tumors using immunohistochemistry and hematoxylin-eosin staining, and we found that the central necrosis is greater for VEGF165-overexpressing tumors than for VEGF189-overexpressing tumors. This indicates that despite having similar total tumor volumes, the viable part of the tumor is larger in VEGF189- than in VEGF165-overexpressing tumors. This also implies that VEGF189-induced microvessels function better than VEGF165-induced microvessels in maintaining the viability of tumors.

Clinically, an association has been found between the expression of different VEGF isoforms in human tumors and different clinicopathological characteristics and patient outcome. Tokunaga *et al.* [14], [41] demonstrated that in colon cancer patients, tumors with VEGF189 mRNA expression have a higher incidence of liver metastasis, higher involvement of the veins, and are associated with a poorer patient prognosis than those lacking VEGF189 mRNA expression. In our own study [15] and others [14]-[16], [41], [42], a high tumor expression of VEGF189 was significantly correlated with large tumors, advanced clinical stage, and systemic metastasis, and was an independent prognosis factor in colorectal, renal cell, and non-small-cell lung cancers. In addition, Tokunaga *et al.* [17] showed that VEGF189 expression is associated with the increased xenotransplantability of human esophageal cancer in the skin of mice. In the present study, the DCE-MRI revealed a high perfusion and permeability function of the tumor microvessels, and SSCE-MRI revealed a very high density of small microvessels penetrating from the tumor rim to the core in VEGF189-overexpressing tumors. These characteristics of tumor angiogenesis may provide a good supply of nutrients and O_2_ for tumor growth, and the highly permeable, dense microvessels may facilitate cancer cell metastasis. These specific functional and structural characteristics of VEGF189-induced tumor angiogenesis may contribute to the reported increased tumorigenesis, high incidence of systemic metastasis, and adverse prognosis of patients and the high xenotransplantability of human cancers that overexpress VEGF189 [14]–[17], [41], [42].

One traditional method used to evaluate tumor angiogenesis is a histological technique called MVD counting in sections of tumor specimens. However, MVD measurements can vary within the tumor, and this may allow sampling bias to produce misevaluations of angiogenesis. Currently, the biomedical imaging modalities that have been used in evaluating tumor angiogenesis include MRI, positron emission tomography, ultrasound, optical imaging, and computed tomography [30], [43]–[45]. Among these, MRI has the advantage of being noninvasive, nonradioactive, rapid**,** and having a high resolution. Our study showed that DCE-MRI is a good method for evaluating the perfusion and permeability functions of tumor microvasculature and that SSCE-MRI is a highly sensitive method for measuring vessel density and size. In this study, DCE- and SSCE-MRI provided a noninvasive and high-resolution imaging method for the evaluation of function and vessel density of tumor angiogenesis *in vivo* and can be performed repeatedly to assess the progress of tumor angiogenesis, and can provide information about the temporal vascular function and spatial distribution of microvessels. These unique characteristics make DCE- and SSCE-MRI useful for assessing tumor angiogenesis before, during, and after anticancer and antiangiogenesis therapies.

We conclude that different VEGF isoforms can induce tumor angiogenesis with different functional and structural characteristics, as demonstrated by DCE- and SSCE-MRI, in non-small-cell lung cancers. VEGF189 can induce the most-dense, small, sprouting microvessels that penetrated deeply from the tumor rim to its core, and has the highest microvessel perfusion and permeability functions. These microvessel characteristics in VEGF189-overexpressing tumors may contribute to the reported adverse effects of VEGF189 overexpression on tumorigenesis, systemic metastasis, and patient survival in several human malignancies, including non-small-cell lung cancer. The finding of high MVD and high perfusion function in VEGF189-induced angiogenesis also has valuable therapeutic implications, suggesting the necessity of aggressive treatment of VEGF189-overexpressing lung cancers.

## Materials and Methods

### Cell lines

A series of model cell lines of a human lung adenocarcinoma (i.e., CL1-0, CL1-1, CL1-5, and CL1-5-F4) with increasing invasiveness and metastatic capabilities was established previously [46], [47]. The CL1-0 cell line, which has low capacities for both tumorigenesis and metastasis and minimal endogenous VEGF production, was chosen for this study.

### Construction of the VEGF isoform expression vector and stable transfection

The full-length cDNA sequence for human VEGF was obtained from a National Center for Biotechnology Information nucleotide sequence search (GenBank E14233, Accession no. NM_001025366). The forward primer, 5′-CGC GGA TCC GAA ACC ATG AAC TTT CTG CTG TCT T-3′ with a BamH1 restriction site, and the reverse primer, 5′-GCT CTA GAC CCG GCT CAC CGC CTC GGC TT-3′ with an Xbal restriction site, were designed, and the PCR products of the VEGF isoforms from normal lung tissue mRNA were purified and verified in sequence, and were used to transfect competent cells.

The cDNA for VEGF121, VEGF165, or VEGF189 was cloned into the constitutive mammalian expression vector pTR2 (BD, Franklin Lakes, NJ, USA) to yield the pTR2-“VEGF isoform constructs”. CL1-0 cells were transfected with the pTR2-VEGF isoform constructs using the Lipofectamine 2000 reagent (Invitrogen, Carlsbad, CA, USA).

### VEGF isoform mRNA and protein expression

The expression of VEGF isoform proteins by CL1-0 cells transfected with the pTR2-VEGF isoform constructs was confirmed by Western blot analysis using rabbit polyclonal anti-human-VEGF as a primary antibody (1∶2000 dilution; Upstate Biotechnology, Waltham, MA, USA), as described previously [13]. VEGF isoform mRNA in the transfected CL1-0 cells or protein in the supernatant was also measured by a real-time quantitative RT-PCR assay (ABI, Carlsbad, CA, USA) [48] and ELISA assay (R&D, Minneapolis, MN, USA), respectively [13].

### *In vivo* tumorigenesis assay in a murine xenograft model

Twenty-four male, 6-week-old severe combined immunodeficiency (SCID) mice from the Laboratory Animal Center, National Taiwan University College of Medicine were used for experiments. For tumor growth in animals, 4×10^6^ control cells (mock) or individual VEGF-isoform-transfected CL1-0 cells in a volume of 0.2 mL were injected subcutaneously into the right dorsal region of each animal, and the mice were examined every 7days (from week 1 to week 5) for tumor appearance by MRI. After 36 days, the animals were sacrificed and the tumors were confirmed by histological examination. The tumor tissue was embedded in OTC medium and frozen at –80°C, and then three consecutive sections were cut from each OTC block for immunohistochemical staining for CD31. The experiments were performed on four groups of six mice each for CL1-0 cells transfected with different VEGF isoforms or mock transfected. The animal experiments were approved by the Laboratory Animal Center, National Taiwan University College of Medicine.

### MRI of the tumor xenografts in SCID mice

All MRI experiments were performed on a horizontal 7.0-T Pharmascan 70/16 spectrometer, and T2-weighted imaging (T2WI) and DCE-MRI were performed on days 7, 14, 21, 28, and 35 after tumor implantation. The mice were initially anesthetized with 5% isoflurane at 5 L/min O_2_. When fully anesthetized, the animal was placed in a prone position and fitted with a custom-designed body holder inside the magnet. Isoflurane was then maintained at 0.5–1.5% at 1 L/min O_2_ throughout the experiments. To maintain body temperature at 37°C, warm air was pumped through the bore of the magnet. Images were acquired using a 38-mm volume coil as both the transmitter and receiver coil. Contrast medium was injected via orbital veins for acquiring DCE- and SSCE-MRI data.

#### 1. Tumor volume measurement

The tumor region was identified from body tissues using T2WI. Multislices of T2WI were acquired by spin-echo sequencing with a repetition time (TR) of 5000 ms, an echo time (TE) of 70 ms, a field of view (FOV) of 3 cm, a number of excitations (NEX) of 2, a matrix size of 256×128 (zero filled to 256×256), and a slice thickness (Slth) of 0.5 mm with no interslice gap so that the entire tumor was covered. The outline of the tumor was delineated by the operator based on the good contrast provided by T2WI between the body tissue and the tumor area. Once the whole tumor region was identified, the total tumor volume was calculated by summing the tumor area in three dimensions using Avizo software (TGS, San Diego, CA, USA).

#### 2. DCE-MRI

The perfusion and permeability functions of tumor angiogenesis were assessed using the *K* ^trans^ map obtained from DCE-MRI using a T1-weighted spin-echo sequence. Spin echo sequence was used because it's insensitive to signal loss and susceptibility artifact in heterogeneous tumor tissue and blood leak; it also provides a better signal-to-noise ratio to increase accurate data fitting [49]. The images were acquired with TR, TE, FOV, Slth, NEX, and matrix-size values of 400 ms, 10 ms, 3 cm, 1.5 mm, 1, and 256×64 (zero filled to 256×256), respectively. A series of 40 axial T1-weighted images were acquired before, during, and after an injection of Gd-DTPA (Magnevist, MW: 938 Dolton, Schering, Berlin, Germany) at a dose of 0.2 mmol/kg. Precontrast T1-weighted maps were acquired by inversion-recovery fast imaging with a steady-state precession sequence, with TR, TE, FOV, Slth, NEX, flip angle, and matrix-size values of 4 ms, 1.8 ms, 3 cm, 1.5 mm, 1, 60°, and 128×128, respectively, and 18 inversion times, ranging from 62 to 2510 ms with an increased interval of 144 ms. The *K* ^trans^ maps were evaluated on days 7, 14, 21, 28, and 35 after tumor implantation.

#### 3. SSCE-MRI

Transverse relaxation rates (ΔR_2_ and ΔR_2_*), obtained by SSCE-MRI, were used to determine the structural properties of the tumor vasculature on day 36 after tumor implantation. ΔR_2_ and ΔR_2_* were determined by performing T2WI and T2*-weighted imaging (T2*WI), respectively, before and after an injection of iron oxide (Resovist, particle size ranged from 45–60 nm, Schering) at a dose of 10 mg/kg. The postcontrast image acquisition was delayed by 5 min to ensure a steady-state distribution of the contrast agent in the vascular network. The O_2_ gas for pumping vaporized isoflurane was used to raise the ΔR2* contrast in veins and venules due to the deoxyhemoglobin within the vessels minimized the difference between precontrast and postcontrast T2*WI. T2WI and T2*WI were performed in the same location with FOV, Slth, NEX, and matrix-size values of 3 cm, 1.5 mm, 2, and 256×128 (zero filled to 256×256), respectively. T2WI was conducted using a fast spin-echo sequence with a TR of 5000 ms, a pseudoecho time of 70 ms, and an echo-train length of 8, while T2*WI was conducted using a fast low-angle shot sequence (FLASH) with a TR of 700 ms, a TE of 10 ms, and a flip angle (FA) of 15°. In FLASH sequence, the contrast depends on the selected parameters of TR, TE, and FA. The FA of 15° with TR of 700 ms was set to reduce T1 weighting on the images, while 10 ms for TE was found to enhance T2* contrast with no susceptibility artifact.

#### 4. Data analysis

In DCE-MRI, the kinetic analysis of dynamic signal enhancement by Gd-DTPA was based on the compartment model of Tofts [20], [21]. The change in the image intensity of DCE-MRI in the tissue was due to leakage of the contrast agent into the tissue from the blood vessel. The rate of contrast agent uptake (d*C*t(*t*)/d*t*) can be given as d*C*t(*t*)/d*t*  =  *K* ^trans^ × (*C*p–*C*t/*v*_e_), where *K* ^trans^ is vascular permeability, *C*p is the concentration of contrast agent in the plasma space, *C*t is the concentration of contrast agent in the tissue extravascular and extracellular space, and *v*_e_ is the leakage space per unit volume of measured tissue. Optimal values of the pharmacokinetic parameters *K* ^trans^ and *v*_e_ for each pixel were calculated by fitting the enhanced curve of the tissue signal using nonlinear regression analysis. In SSCE-MRI, the transverse relaxation rate shifts are given by ΔR_2_  =  ln(*S*_pre_/*S*_post_)/TE and ΔR_2_^*^  =  ln(*S*_pre_^*^/*S*_post_^*^)/TE, where *S*_pre_, *S*_post_ and *S*_pre_^*^, *S*_post_^*^ are the precontrast and postcontrast signal intensities for the spin echo and gradient echo, respectively. The rVDI and rVSI are derived from the relaxation rate shift ratio as ΔR_2_/(ΔR_2_*)^2/3^ and ΔR_2_*/ΔR_2_, respectively [22]–[26]. All of the parametric maps (*K* ^trans^, rVSI, and rVDI) were analyzed in the regions of the tumor core and rim, which were delineated by T2WI. The region of the tumor core was defined as 50% of the distance from the central point of tumor to its outside boundary, while the region of tumor rim was determined by the subtraction of the area of the tumor core from that of the total tumor area (Fig. 2). The processing software for all of the quantitative analyses of the data obtained by DCE- and SSCE-MRI was written in MATLAB (MathWorks, Natick, MA, USA).

### Assessment of phenotypes of tumor angiogenesis and microvessel counts by immunohistochemical staining

The resected tumor specimens were embedded in OTC medium and frozen at –80°C. Sections (5 µm) were then prepared and stained for 60 min at room temperature with rat monoclonal anti-mouse CD31 antibody (1∶20 dilution; PharMingen, San Diego, CA, USA) to stain endothelial cells. The sections were washed and then incubated for 30 min at room temperature with rabbit anti-rat Ig antibody (Zymed Laboratories, South San Francisco, CA, USA), and then for a further 30 min at room temperature with a 1∶20 dilution of avidin-biotin peroxidase complex (Zymed Laboratories). The color was developed by incubating the slides for 20 min with 3,3-diaminobenzidine (Zymed Laboratories). Counterstaining was performed using Mayer's solution, giving a blue background. The capillaries surrounding the alveoli of normal mouse lung tissue were used as the positive control for anti-CD31 antibody staining. Negative controls were sections stained without the use of primary antibodies or using a control IgG instead of primary antibodies. The density and size of the vasculature were analyzed in digital images using the Metamorph imaging processing package (Universal Imaging Corporation, West Chester, PA, USA).

### Statistical analysis

All statistical tests were performed using StatView software (version 5.0.1, SAS Institute, Cary, NC, USA). Comparison of variables (temporal changes in tumor volume and *K* ^trans^) among tumors overexpressing different VEGF isoforms and the mock-transfected cells was performed using two-way ANOVA analysis followed by Fisher's *post hoc* test for pair comparisons. One-way ANOVA analysis followed by Fisher's *post hoc* test for pair comparisons was used to investigate the effects of different VEGF isoforms on rVDI, rVSI, MVD, and microvessel size. Data are presented as mean±SD values, and the level of statistical significance was set at *p*<0.05.
